# Amyloid β Peptide Enhances RANKL-Induced Osteoclast Activation through NF-κB, ERK, and Calcium Oscillation Signaling

**DOI:** 10.3390/ijms17101683

**Published:** 2016-10-10

**Authors:** Shangfu Li, Bu Yang, Dian Teguh, Lin Zhou, Jiake Xu, Limin Rong

**Affiliations:** 1 Department of Spine Surgery, The Third Affiliated Hospital of Sun Yat-sen University, TianHe Road 600, TianHe District, Guangzhou 510630, China; lishangfu2013@163.com (S.L.); xinfeibbb@gmail.com (B.Y.); 2 Molecular Lab, School of Pathology and Laboratory Medicine, University of Western Australia, Perth 6009, WA, Australia; dian.teguh@research.uwa.edu.au (D.T.); linzhou09250600@gmail.com (L.Z.); jiake.xu@uwa.edu.au (J.X.)

**Keywords:** osteoporosis, amyloid β peptide, osteoclast, NF-κB, MAPK, NFAT-c1

## Abstract

Osteoporosis and Alzheimer’s disease (AD) are common chronic degenerative disorders which are strongly associated with advanced age. We have previously demonstrated that amyloid beta peptide (Aβ), one of the pathological hallmarks of AD, accumulated abnormally in osteoporotic bone specimens in addition to having an activation effect on osteoclast (Bone 2014,61:164-75). However, the underlying molecular mechanisms remain unclear. Activation of NF-κB, extracellular signal-regulated kinase (ERK) phosphorylates, and calcium oscillation signaling pathways by receptor activator NF-κB ligand (RANKL) plays a pivotal role in osteoclast activation. Targeting this signaling to modulate osteoclast function has been a promising strategy for osteoclast-related diseases. In this study, we investigated the effects of Aβ on RANKL-induced osteoclast signaling pathways in vitro. In mouse bone marrow monocytes (BMMs), Aβ exerted no effect on RANKL-induced osteoclastogenesis but promoted osteoclastic bone resorption. In molecular levels, Aβ enhanced NF-κB activity and IκB-α degradation, activated ERK phosphorylation and stimulated calcium oscillation, thus leading to upregulation of NFAT-c1 expression during osteoclast activation. Taken together, our data demonstrate that Aβ enhances RANKL-induced osteoclast activation through IκB-α degradation, ERK phosphorylation, and calcium oscillation signaling pathways and that Aβ may be a promising agent in the treatment of osteoclast-related disease such as osteoporosis.

## 1. Introduction

Osteoporosis is an age-related public health issue characterized by low bone mass and increased susceptibility to fracture. Although osteoporosis is a major health burden in the elderly, treatments for this disease are far from ideal. Osteoporosis and Alzheimer’s disease (AD) are common chronic degenerative disorders which are strongly associated with advanced age [1,2,3]. AD is frequently associated with fractures and reduction in bone mineral density, even at early stages of disease when patients are still active [4,5,6], indicating that AD and osteoporosis may share conserved pathogenic mechanisms. We have previously shown that there is a significant elevated expression levels of amyloid β peptide (Aβ), one of the pathological hallmarks of AD, in osteoporotic bone tissues. Moreover, Aβ also enhances osteoclast (OC) function [7]. Previous studies have identified a role for Aβ in the activation of OC through gene knockout experiments and the use of transgenic AD mouse model, Tg2576 [8,9]. However, the underlying molecular mechanisms associated with OC activation by Aβ are still elusive.

OCs are monocyte/macrophage lineage-derived large multinucleated cells that play a pivotal role in bone remodeling [10,11]. Macrophage colony stimulating factor (M-CSF) and receptor activator of nuclear factor κB ligand (RANKL) are two essential factors in the differentiation and activation of OC [12]. M-CSF stimulates the proliferation and survival of OC precursors and mature cells. RANKL, however, is the primary OC differentiating factor which exerts its functions through initiating downstream signaling by activating its receptor RANK, which in turn activates nuclear factor κB (NF-κB), mitogen-activated protein kinases (MAPK) and calcium oscillation [13]. It is well recognized that NF-κB activation is among the very early signaling events which plays an important role during osteoclastogenesis [14]. Calcium signaling in OC is essential for diverse cellular functions including differentiation, bone resorption, and gene transcription [15]. RANKL recruits TNF receptor associated factors (TRAFs) to activate the phosphorylation of extracellular signal-regulated kinase (ERK, one of a well-known MAPK), which subsequently forms activator protein 1 (AP-1) complexes with c-Fos, an essential transcription factor for osteoclastogenesis [16]. Accumulating evidence suggests nuclear factor of activated T cells (NFATc1) is upregulated by RANKL in OC precursors through mechanisms that depend on NF-κB and AP-1, which can directly regulate a number of OC-related marker gene expression [17], including tartrate resistant acid phosphatase (TRAP), calcitonin receptor (CTR), and cathepsin K which were previously demonstrated in our study by RT-PCR [7]. Therefore, targeted modulation of these signaling pathways to regulate NFATc1 expression may be helpful for the treatment of OC-related diseases such as osteoporosis.

Our study aims to verify the hypothesis that Aβ may have an activation effect on OC function through NF-κB, ERK phosphorylation, and calcium signaling pathways in vitro.

## 2. Results

### 2.1. Aβ42 Exerts No Effect on RANKL-Induced Osteoclastogenesis but Enhances Osteoclastic Bone Resorption

**Figure 1:**
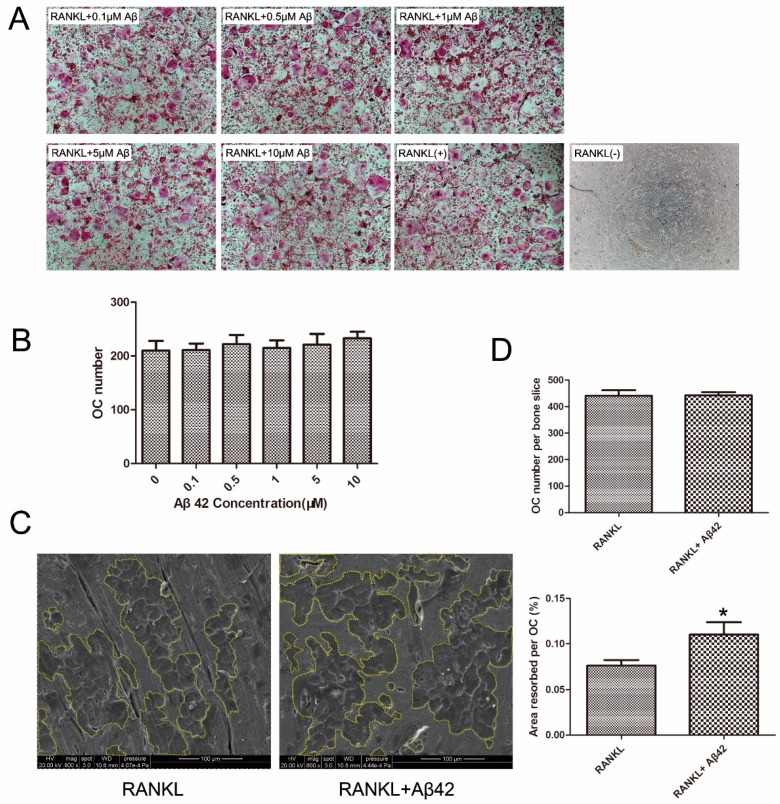
Aβ42 has no effect on RANKL-induced osteoclastogenesis but enhances osteoclast (OC) function. (**A**) Light microscopy images depicting the effect of various concentrations (0.1, 0.5, 1, 5, 10 μM) of Aβ42 on RANKL-induced OC formation at 40× magnification positively stained with tartrate resistant acid phosphatase (TRAP). TRAP positive cells containing three or more nuclei were counted as OCs; (**B**) Quantification of mature OC showing no statistical difference among cells treated with different concentrations of Aβ42; (**C**) Representative scanning electron microscopy (SEM) images of resorption area in bone slices showing Aβ42 enhanced RANKL-induced bone resorption by OCs; (**D**) Quantification analysis showing Aβ42 enhanced OC activation by increasing resorption area (~1.6-fold change). Bar in C represents 100μm. Experiments were carried out in triplicate and results are presented as mean ± SD. * *p* < 0.05.

To examine the effect of Aβ42 on RANKL-induced osteoclatogenesis, mouse bone marrow monocytes (BMMs) cultures were incubated with various concentrations of Aβ42 and evaluated for formation of OCs. Unexpectedly, no obvious differences in mature OC numbers were observed, as shown in the representative images of OC precursors treated with five concentrations (0.1/0.5/1/5/10 μM) of Aβ42 compared with the positive control (RANKL(+)) (Figure 1A,B). To test the effects of Aβ42 on osteoclastic bone resorption (OC activation), equal numbers of pre-OC cells were seeded onto bone slices and Aβ42 was added to cultures following cell attachment. Bone slices were retrieved on day 9, after culture with Aβ42 and RANKL treatment. These bone slices were analyzed using scanning electron microscopy (SEM) and the area of resorption pits were measured. Treatment of cultures with Aβ42 enhanced osteoclastic bone resorption (Figure 1C,D). Note that treatment of Aβ42 showed more resorption pits and larger pit areas compared to the untreated control (Figure 1C). Importantly, this increase in bone resorption area was not due to OC cell death as no significant differences in the total number of TRAP-positive cells per bone slice were observed after the addition of Aβ42 (Figure 1D). Collectively, this result indicates that Aβ42 does not affect RANKL-induced osteoclastogenesis but promotes osteoclastic bone resorption.

### 2.2. Aβ42 Enhances RANKL-Stimulated NF-κB Activity and IκB-α Degeneration

RANKL-induced NF-κB activation is essential in initiating OC differentiation and function [14]. The effects of Aβ42 on RANKL-induced signaling transduction pathways were examined to gain insights into the molecular mechanisms underlying its actions on OC differentiation and activity.

**Figure 2:**
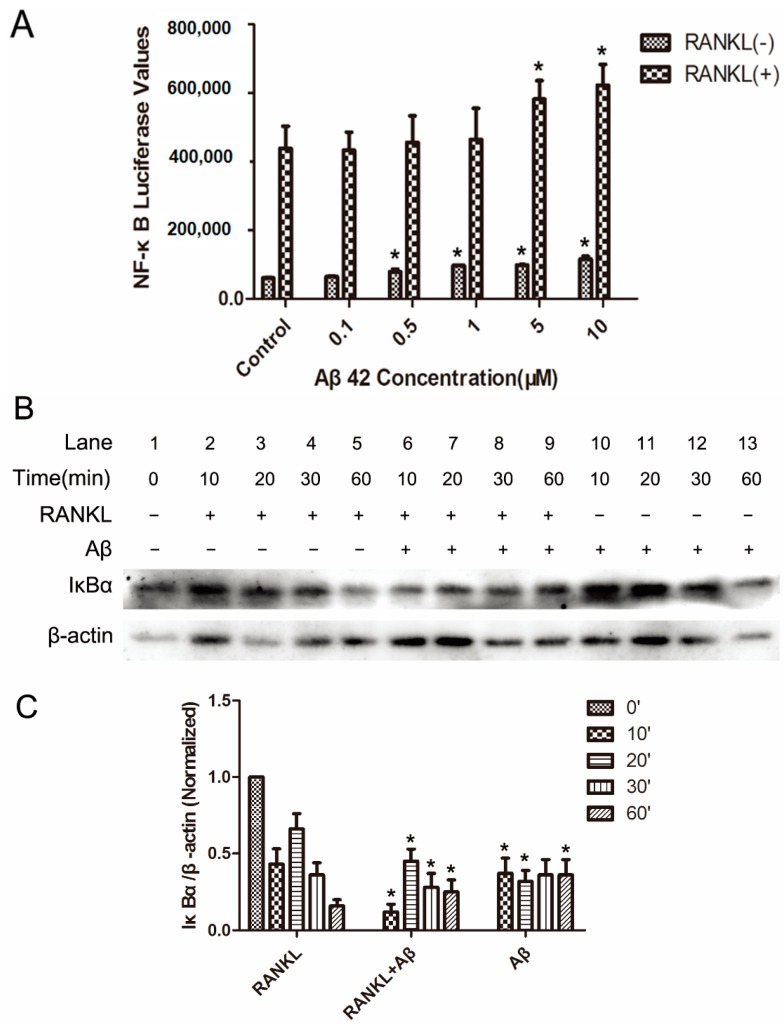
Aβ42 enhances RANKL-stimulated NF-κB activity and IκB-α degeneration. (**A**) RAW264.7 cells stably transfected with NF-κB transcriptional luciferase reporter construct, were pretreated with Aβ42 for 1 h and then stimulated with 50 ng/mL RANKL or treated with Aβ42 alone for a further 6 h, followed by luciferase activity quantification. * *p* < 0.05 vs. control group; (**B**) Representative blots of mouse bone marrow monocytes (BMMs) lysates for IκB-α and β-actin (*n* = 3). BMMs pretreated with Aβ42 at 5 μM for 1 h were treated with 50 ng/mL RANKL for 0, 10, 20, 30, and 60 min as indicated; (**C**) Average ratio of IκB-α relative to β-actin. ′ in (**C**) stands for minute. Western blot signal intensities were quantified using Image J. IκB-α/β-actin ratios were normalized to 0 min, results are presented as mean ± SD. * *p* < 0.05 vs. control group with RANKL treatment alone at the corresponding time point.

The effect of Aβ42 on RANKL-induced NF-κB activity in OC precursors was determined by using luciferase assay. RANKL induced an eight-fold increase in the NF-κB dependent luciferase activity compared to the unstimulated cells. Aβ42 had a modest effect on this signal induction, with 5 μM showing approximately 25% increases and 10 μM showing approximately 30% enhancement in NF-κB activation (Figure 2A). Treatment of Aβ42 alone showed a trend of increase in the basal level of NF-κB activity in BMMs at 0.5, 1, 5, and 10 μM (Figure 2A). However, it was much weaker compared to treatment of RANKL alone. The primary regulatory point of NF-κB activity is at the level of IκB protein degradation. Consistent with the results of NF-κB luciferase assay, further examination by western blotting showed that, in BMMs treated with RANKL, RANKL stimulation led to the phosphorylation and ~50% degradation of IκB-α within 10 min; 5 μM Aβ42 treatment did increase IκB-α degeneration in 10 min (Figure 2B, lane 6 compared to lane 2), 20 min (Figure 2B, lane 7 compared to lane 3) and 30 min (Figure 2B, lane 8 compared to lane 4) when compared with the control groups (Figure 2C). It is noteworthy that Aβ42 alone had a similar but smaller effect on IκB protein degradation in 10 min (Figure 2B, lane 10 compared to lane 2) and 20 min (Figure 2B, lane 11 compared to lane 3) when compared with the RANKL-only-induced group (Figure 2C). All these results indicate that Aβ42 enhances RANKL-mediated activation of NF-κB signaling pathways by increasing IκB-α degradation in OC.

### 2.3. Aβ42 Promotes the Phosphorylation of ERK Induced by RANKL

Previous studies have indicated that RANKL induces the downstream activation of ERK, which is essential for OC activation [16,18]. Thus, we determined whether ERK activation was involved in the enhancement of Aβ42 on OC function.

**Figure 3:**
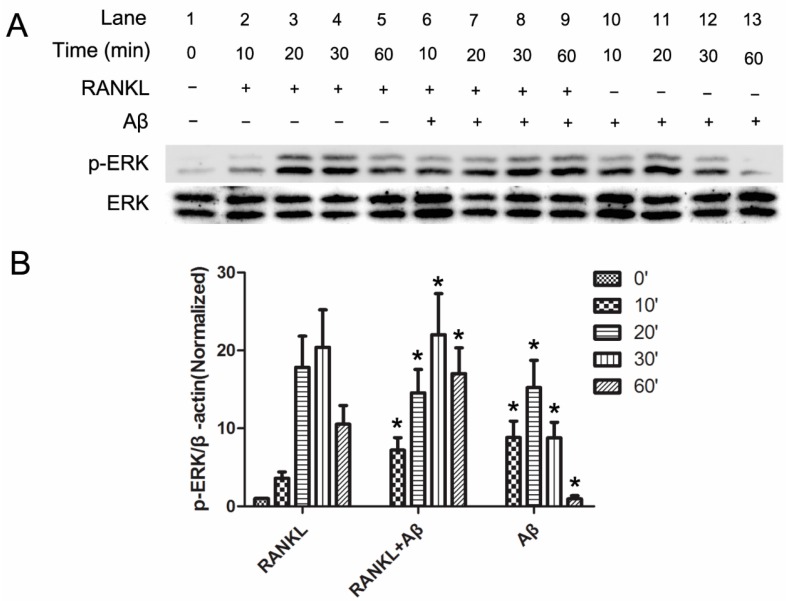
Aβ42 induces the phosphorylation of ERK. (**A**) BMM cells were starved in serum free media for 4 h and incubated with or without Aβ42 (5 μM) in the last hour, followed by treatment with RANKL (50 ng/mL) for the indicated time points. Cell lysates were subjected to immunoblotting and activation of signaling molecules were evaluated using phosphorylated antibody against ERK; (**B**) Average ratio of p-ERK relative to β-actin. Western blot signal intensities were qualified by Image J. p-ERK/β-actin ratios were normalized to 0 min, results are presented as mean ± SD. ′ in (**B**) stands for minute. * *p* < 0.05 vs. control group with RANKL treatment alone at the corresponding time point.

Mouse BMM cells were incubated with serum free medium for 4 h, 5 μM Aβ42 was added in the last hour. Cells were then stimulated with 50 ng/mL RANKL for the following time points: 0, 10, 20, 30, and 60 min. Cells were lysed and subjected to western blot (WB) analysis. Phosphorylation of ERK was observed at 10 min after RANKL treatment in BMMs and it was enhanced by pre-treatment with Aβ42 (Figure 3A, lane 6 compared to lane 2, p-ERK/ERK ratio = 7.22 to 3.61). Moreover, WB analysis showed elevated p-ERK expression levels in 30 min (Figure 3A, lane 8 compared to lane 4, *p*-ERK/ERK ratio = 22.00 to 20.42) and 60 min (Figure 3A, lane 9 compared to lane 5, p-ERK/ERK ratio = 17.05 to 10.54) after adding Aβ42 when compared with the control groups of RANKL treatment alone (Figure 3B). Interestingly, a similar effect of ERK phosphorylation induced by RANKL during OC activation was found when using Aβ42 alone. ERK phosphorylation induced by Aβ42 alone appeared in 10 min after incubation (Figure 3A, lane 10) and reached its peak in 20 min (Figure 2, lane 11), then decreased gradually in 30 min (Figure 3A, lane 12), and almost disappeared in 60 min (Figure 3A, lane 13). However, the induction of Aβ42 alone on ERK phosphorylation was smaller when compared with the RANKL treatment only group (Figure 3B). Accordingly, these results indicated that Aβ42 enhanced ERK phosphorylation with a similar trend but smaller induction compared to RANKL during OC differentiation.

### 2.4. Aβ42 Increases RANKL-Induced Calcium Oscillation during OC Differentiation

As calcium signaling plays a critical role in the differentiation and functions of OC [15], where cytoplasmic Ca^2+^ oscillations occur during RANKL-mediated osteoclastogenesis; we proceed to examine the effects of Aβ42 on calcium oscillation in OC.

Mouse BMMs were seeded on a culture plate and incubated overnight to ensure cell attachment before 2 h pre-treatment with 1 or 5 μM Aβ42, followed by treatment with 50 ng/mL RANKL for 24 h. Cells without Aβ42 treatment were used as control. Cells were washed with assay buffer and calcium oscillations were examined using Fluo4 stain observed under fluorescence microscopy. The 20 brightest cells in each group were selected and analyzed. Consistent with the enhancement of Aβ42 on NF-κB activity and ERK phosphorylation (Figure 1 and Figure 2), Table 1 showed the intensity of calcium oscillations following RANKL stimulation was significantly increased on Aβ42 treated BMM cells (RANKL + Aβ groups) compared to untreated BMM cells (RANKL only group). As expected, treatment with RANKL induced calcium oscillations whereas no calcium flux was observed in the RANKL-untreated group. Treatment with Aβ42 significantly enhanced RANKL-induced calcium oscillations. The oscillation intensity became stronger in the RANKL + 5 μM Aβ42 group (238 ± 47 to 189 ± 45/cell) and oscillation frequency became quicker in both the RANKL + 1 μM Aβ42 group (8 ± 3 to 6 ± 2 times/min) and RANKL+5 μM Aβ42 group (9 ± 2 to 6 ± 2 times/min) when compared with the RANKL-only control. Importantly, treatment with Aβ42 alone showed a trend of increase in calcium oscillation intensity (98 ± 46 to 8 ± 6/cell) at 5 μM, compared to the control. However, it was much weaker when compared with treatment of RANKL alone (98 ± 46 to 189 ± 45/cell). These results suggest Aβ induces the calcium signaling mediated by RANKL.

Quantitative analysis of the average change in oscillation intensity per cell and frequency per minute showing calcium oscillation pattern in mouse BMM cells stimulated with RANKL in the present or absent of Aβ42. Cells pretreated with 1 and 5 μM Aβ42 for 1 h prior to stimulation with 50 ng/mL RANKL, and control cells received M-CSF only. Calcium fluctuations were analyzed across multiple cells (*n* = 20 individual cells/group, three wells/treatment) for each condition and maximum peak intensity/frequency minus baseline intensity/frequency was calculated (*n* = 3). * *p* < 0.05 vs. RANKL-stimulated group.

### 2.5. Aβ42 Enhances NFAT Activity and Protein Expression of NFATc1 during OC Differentiation

NFATc1 is the master regulator of OC development. Calcineurin/NFAT transcriptional axis is essential for OC bone resorption [17]. Although the transcriptional activity of NFATc1 cannot be distinguished from NFATc2, NFATc1 is the crucial factor induced by RANKL and both autoamplify their own transcription and that of NFATc2 during RANKL-mediated osteoclastogenesis [19].

**Figure 4:**
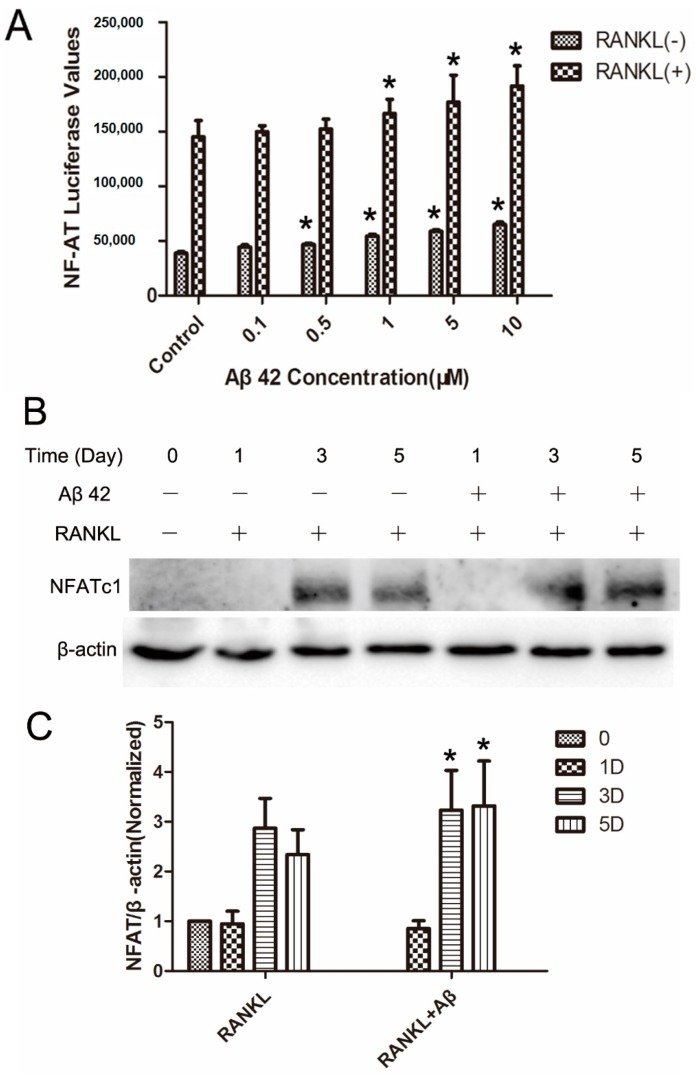
Aβ42 enhances NFAT activity and protein expression of NFATc1 during osteoclast differentiation. (**A**) RAW264.7 cells stably transfected with a NFAT transcriptional luciferase reporter construct, were pretreated with Aβ42 (0.1, 0.5, 1, 5, and 10 μM) for 1 h and then stimulated with 50 ng/mL RANKL for 24 h (*n* = 3). * *p* < 0.05 vs. control group; (**B**) Representative blots of cell lysates for NFATc1 and β-actin expression. BMM cells were pretreated with 5 μM Aβ42 for 1h prior to 100 ng/mL RANKL stimulation for 0, 1, 3, and 5 days; (**C**) Average ratio of NFAT relative to β-actin. Western blot signal intensities were quantified using Image J. NFAT-c1/β-actin ratios were normalized to 0 day, results are presented as mean ± SD. “D” in C stands for Day. * *p* < 0.05 vs. control group with RANKL treatment only at the corresponding time point.

The effect of Aβ42 on RANKL-induced NFATc1 activity in OC precursors was examined using luciferase assay. RANKL induced an almost four-fold increase in the NFATc1 dependent luciferase activity compared to the unstimulated cells. Aβ42 had a modest effect on this signal induction, with 1 μM showing approximately 10% increases, 5 μM showing approximately 20% enhancement and 10 μM showing approximately 30% enhancement in signaling (Figure 4A). More importantly, treatment of Aβ42 alone showed a trend of increase on the basal level of NFATc1 activity in BMMs at 0.5, 1, 5, and 10 μM (Figure 4A). However, it is much weaker compared to the treatment of RANKL alone. To further examine the effects of Aβ42 on NFATc1 protein expression, we stimulated the BMM cells for 1, 3, and 5 days with RANKL in the presence or absence of Aβ42 (5 μM). Consistent with the luciferase assay, WB analyses showed that Aβ42 at 5 μM moderately enhanced RANKL-induced NFATc1 levels at day 3 (Figure 4B, ~1.2-fold change) and day 5 (Figure 4B, ~1.4-fold change) when compared with the RANKL-only-induced group (Figure 4C).

Collectively, these results indicated that Aβ42 could enhance RANKL-mediated activation of NF-κB, ERK phosphorylation, and calcium oscillation signaling pathways during OC differentiation and function.

## 3. Discussion

In this study, we found that treatement of Aβ42 enhanced NF-κB activation and IκB-α degradation increased phosphorylation of ERK in primary mouse BMMs during OC differentiation and activation. Furthermore, our work suggests that Aβ42 activated RANKL-induced calcium oscillation in OC. Consistent with these results, Aβ42 enhanced NFAT activity and protein expression of NFATc1, which promotes osteoclastic bone resorption, with no effect on OC formation. Hence, these data are consistent with our hypothesis that Aβ42 enhanced RANKL-induced OC activation through NF-κB, MAPK, and calcium oscilation signaling pathways. To the best of our knowledge, these results show the effects of Aβ activation signaling pathways on OC for the first time. Moreover, this work confirmed our previous study [7] in a deeper molecular levels, indicating that Aβ may have an important role in the pathogenesis of osteoporosis and that Aβ42 may be considered as a novel therapeutic target in the preventive strategy against osteoporosis.

What is the exact molecular mechanisms underlying OC activation by Aβ42? Cui et al. demonstrated that Aβ mediated OC differentiation and function by the Receptor for Advanced Glycation End-products (RAGE). RAGE is a member of the immunoglobulin superfamily with multiple ligands, which is crucial for the pathogenesis of diverse disorders [20,21] in Tg2576 mice [8,9], leading to the convergence of α_γ_β_3_ integrin- and M-CSF-mediated signals including activation of Rac, PYK2, Src, ERK, and c-Fos. These findings are consistent with our results on the ERK phosphorylation and NF-κB signaling pathways. Given that calcium oscillation signaling is essential for OC differentiation, gene transcription, and bone resorption [15,22], and that activated calcium signaling promotes Aβ formation and the amyloid pathology of AD [23], these intersections lead us to explore and proved eventually that Ca^2+^ pathways also play a critical role in Aβ-mediated OC differentiation and activation. Together, these signaling cascades may initiate the common pathway of NFATc1, a master regulator of OC activation, expression, and translocation into the nucleus, which eventually induces OC-specific gene transcription to enhance OC function. Nevertheless, the changes in the activation of NF-κB, ERK, and calcium oscillation signaling are not significant in every time point in our study, although obvious effects on OC activation (osteoclastic bone resorption) were observed after Aβ treatment. We speculate that these signals may cause a positive feedback and it will be amplified after activation. Moreover, we postulate that the three signaling pathways may have synergistic effect on OC formation and bone resorptive activity, thus leading to OC activation in a convergent manner.

Osteoclasts, bone-specified multinucleated cells, are responsible for bone destructive diseases such as osteoporosis [24]. The gene expression of TRAP, cathepsin K, and CTR is increased during osteoclastogenesis which affects OC function and fusion [25]. The enhancement of Aβ on OC function investigated by our previous study [7] thus might be due to its activation of the underlying signaling pathways in OC. In RANKL-induced signalling pathways [26], NF-κB activation is critical for osteoclastogenesis [27]. NF-κB is activated by RANKL both in RAW264.7 cells and BMM cells, and it is required for OC formation [14]. In this study, Aβ was found to enhance the RANKL-mediated NF-κB dependent transcription activity and IκB-α degradation in BMM cells. This suggests that the effect of Aβ on RANKL-induced NF-κB activity may be partly reliant on IκBα degradation, or could simply have a moderate effect on NF-κB activity in these cells. This may also be owning to the secondary effect of Aβ on signalling pathways or other unknown regulatory functions that have an impact on NF-κB. The promotion of RANKL-induced NF-κB activation by Aβ might partly explain the enhancement of Aβ on OC activation [7], however, it is unlikely to be the most important mechanism.

NFATc1, whose overexpression can drive OC formation independently of RANKL, is a critical transcription factor which is essential for osteoclastogenesis [28]. Indeed, we observed that Aβ increased RANKL-induced NFAT activity and NFATc1 levels, which is highly suggestive of an enhancement action of Aβ upon OC commitment. Moreover, NFATc1 levels are regulated by the transcriptional control and the actions of the Ca^2+^-dependent phosphatase calcineurin, which stabilises the protein and allows its nuclear transcription and transcriptional activity [15,29]. Importantly, NFATc1 can autoamplify the transcription of NFATc1 itself and NFATc2 with which it cooperates to bind NFAT response elements in gene promoters such as V-ATPase-d2, cathepsin K, and β3 integrin [17]. We demonstrated that Aβ could enhance the transcription of OC differentiation marker gene of NFATc1 in our previous study [7] and increase NFATc1 activity and protein expression in this study. This indicates that Aβ enhances OC function via an NFATc1-mediated pathway. Since NF-κB and NFATc1 coodinate to promote expression of most osteoclast marker genes [30], Aβ is able to promote the transcription of osteoclast-specific genes such as TRAP and cathepsin K, contributing to the enhancement effect of Aβ on OC function. Consistent with a study showing high extracellular calcium affected osteoclastogenesis in mouse BMM cell culture [31], our results further demonstrate that Aβ enhances RANKL-induced calcium oscilations in BMMs, indicating that Aβ upregulates NFATc1 in response to RANKL stimulation by increasing calcium signaling-dependent NFAT activation.

In summary, our data clearly demonstrate that Aβ enhances RANKL-induced OC activation and function through NF-κB, ERK, and calcium pathways, unveiling an important role for Aβ in the pathogenesis of osteoporosis at molecular levels in OC. Our study may have important implications for the molecular mechanisms regulating osteoporosis and AD. These findings may help healthcare professionals and researchers in developing exciting new potential therapeutic agents aimed towards resorbing OC, particularly those designed by targeting Aβ, to efficiently suppress OC and restore physiological bone mineral density.

## 4. Experimental Section

### 4.1. Reagents

Aβ42 peptide was purchased from GL Biochem (052487, Shanghai, China) and dissolved in 1× PBS. Alpha modified Eagles medium (α-MEM) and fetal bovine serum (FBS) were purchased from ThermoFisher (Los Angeles, CA, USA). Anti-ERK (sc-135900), anti-p-ERK (sc-377400), anti-IκBα (sc-7073), anti-NFATc1 (sc-7294), and anti-β-actin antibodies were purchased from Santa Cruz Biotechnology (Santa Cruz, CA, USA). Recombinant GST-rRANKL protein was expressed and purified as previously described [32] and recombinant macrophage colony stimulating factor (M-CSF, R&D Systems, Minneapolis, MN, USA) was used as previously described [33].

### 4.2. Macrophage Isolation from Mouse Bone Marrow, Osteoclastogenesis Assay, and Tartrate Resistant Acid Phosphatase (TRAP) Staining

BMMs from wildtype C57BL/6J mice were prepared as previously described [33]. The non-adherent fraction of mouse BMMs were cultured in α MEM media supplemented with 10% fetal bovine serum, 1% antibiotic/anti mycotic agent, and 30 ng/mL of recombinant mouse M-CSF. The resulting non-adherent cells represent purified BMMs. BMMs were then cultured in complete MEM-α (10% FBS and 100 U/mL penicillin and 100 μg/mL streptomycin) supplemented with 30 ng/mL M-CSF for three days. The BMMs of passage 1 or 2 were used in the experiments below. The BMMs were then seeded on to 96-well plate (6 × 10^3^ cells/well) and treated with 30 ng/mL M-CSF, 50 ng/mL RANKL with or without Aβ42 at different concentrations (0.1, 0.5, 1, 5, 10 μM). The media was replaced with supplemented MCSF and RANKL with or without Aβ42 every two days to generate OCs. After seven days of culture, the cells were fixed with 2.5% glutaraldehyde and stained with TRAP. The TRAP staining was performed using an Acid Phosphatase staining kit (387A, Sigma-Aldrich, St. Louis, MO, USA) according to the manufacturer’s instructions. TRAP positive cells with three or more nuclei were counted as multinucleated OCs using light microscopy. Experiments were approved by the Institutional Animal Care and Use Committee (Approval No: IACUC-2012-0504) of Sun Yat-sen University.

### 4.3. Functional Lacunar Resorption Pit Formation Assay

The bone-resorbing activity of mature OCs derived from BMM cells was analyzed on bone slices which were placed in the bottom of each well of 96-well plates. BMM cells (6 × 10^3^ cells/well) were cultured in complete α-MEM supplemented with 100 ng/mL RANKL in the presence and absence of 5 μM Aβ42. After nine days of co-culture at 37 °C incubation, the bovine bone slices were then incubated for 2 h in 2 M NaOH. OCs were then removed by mechanical agitation and sonication. Lacunar resorption pits were visualized under Philips XL30 scannning electron microscope (SEM) and the percentage of bone surface area resorbed was quantified using Image-Pro Plus software (Media Cybernetics, Bethesda, MD, USA).

### 4.4. Luciferase Assay

RAW264.7 cells stably transfected with a NF-κB transcriptional luciferase reporter and NFATc1 luciferase reporter were used to measure the activity of NF-κB and NFATc1 respectively [34,35]. Cells were plated at 1.5 × 10^5^ cells/0.2 mL/well in a 48-well plate and left overnight to allow attachment. Cells were pre-treated for 1 h with various concentrations (0, 0.1, 0.5, 1, 5, 10 μM) of Aβ42 and stimulated with or without 50 ng/mL RANKL for 6 h, negative control cells received M-CSF only. Cells were lysed with luciferase lysis buffer (100 μL/well) containing 25 mM Tris HCl pH 7.8, 2 mM EDTA, 10% glycerol, 1% Triton-X 100 and 2 mM DTT. 50 μL sample of the lysates were placed into a clean white-bottomed 96-well plate. Finally, 50 μL of luciferase assay substrate (E1500, Promega, Fitchburg, MA, USA) was added at 1:1 ratio lysates to substrate. The plate was then read using the BMG Polar Star Optima Luminescence reader. Luciferase values were expressed as NF-κB and NFATc1 activity. Experiments were performed in triplicate and results are presented as mean ± SD.

### 4.5. Western Blotting Assay

BMM cells were stimulated with RANKL and M-CSF as above, in the presence or absence of 5 μM Aβ42. Cells were lysed in RIPA buffer containing 20 nM Tris-HCl, 150 mM NaCl, 1% Triton X-100, 0.2% deoxycholate, protease, and phosphatase inhibitors for 30 min on ice for protein extraction. Protein concentrations of cell lysates were determined by using BCA assay. An equal amount of proteins (30 μg/lane) were resolved by SDS-polyacrylamide gel electrophoresis and were then transferred to a PVDF membrane (Millipore, Boston, MA, USA). The membrane was probed with the indicated primary antibodies. Blots were developed using horseradish peroxidase-conjugated secondary antibodies and were visualized by using enhanced chemiluminescence (ECL) reagents (Amersham, Pittsburgh, PA, USA) according to manufacturer’s instructions. Images were acquired on an Imagequant LAS 4000. β-Actin was detected on the same membrane and used as a loading control. Experiments were repeated three times and quantification of all the blots is presented as mean ± SD. Signal intensities were quantified by NIH Image J software.

### 4.6. Measurement of Intracellular Ca^2+^ Oscillation

2.5 × 10^4^ BMM cells were seeded on a 48-well plate and cultured with 100 ng/mL RANKL in the presence of 30 ng/mL M-CSF for 24 h. Treatment cells were pretreated with 5 μM Aβ42 for 1 h prior to stimulation in the present/absent of RANKL, negative control cells received M-CSF only. Cells were incubated with 5 μM fluo-4 AM, and 0.05% pluronic F-127 (Invitrogen, Los Angeles, CA, USA) for 30 min at 37 °C in Hank’s Balanced Salt Solution supplemented with assay buffer containing 1% FCS/1 mM probenecid. Cells were washed twice in assay buffer and incubated at room temperature for another 20 min. The cells were washed again in the assay buffer and finally observed on an inverted fluorescent microscope (Nikon, Melville, NY, USA) at an excitation wavelength of 488 nm. Images were captured at 2 s intervals for a total of 3 min and at 40× magnification. The 20 brightest oscillating cells (at least two oscillations viewed) in each group were selected and analyzed for average peak height (maximum peak intensity/frequency minus baseline intensity/frequency) per well in triplicate wells per treatment by using Nikon Basic Research Software. Two experienced technicians were blinded to the identity of each well at the time they determined the 20 brightest cells and the results were evaluated by independent consensus between them.

### 4.7. Statistical Analysis

Quantitative data was analyzed using ANOVA and Tukey’s post hoc test. All data was collected from at least three independent experiments and was presented as mean ± standard error.

## Figures and Tables

**Table 1:** Aβ42 increases RANKL-induced calcium oscillation.

Groups	Average Change in Oscillation Intensity (/cell)	Average Change in Oscillation Frequency (times/min)
Control	8 ± 6 *	1 ± 1 *
RANKL	189 ± 45	6 ± 2
Aβ (5 μM)	98 ± 46 *	2 ± 2 *
RANKL + Aβ (1 μM)	205 ± 53	8 ± 3 *
RANKL + Aβ (5 μM)	238 ± 47 *	9 ± 2 *

* *p* < 0.05.
